# Matrix Metalloproteinases 1 and 3 in Ovarian Cancer: Diagnostic and Prognostic Potential of Genetic Variants and Expression Profiling

**DOI:** 10.3390/diagnostics15121521

**Published:** 2025-06-15

**Authors:** Amal Mohamad Husein Mackawy, Hajed Obaid Alharbi, Ahmad Almatroudi, Wanian M. Alwanian, Khaled S. Allemailem

**Affiliations:** Department of Medical Laboratories, College of Applied Medical Sciences, Qassim University, Buraydah 51452, Saudi Arabia; hajed.alharbi@qu.edu.sa (H.O.A.); aamtrody@qu.edu.sa (A.A.); w.alwanian@qu.edu.sa (W.M.A.)

**Keywords:** ovarian carcinoma, malignancy risk, cancer progression, prognosis, biomarkers, matrix metalloproteinases, genetics, protein expression of MMPs 1,3, MMP immunohistochemical staining, single-nucleotide polymorphism of MMPs 1,3, polymerase chain reaction

## Abstract

**Background:** Ovarian carcinoma (OC) is one of the foremost factors in female carcinoma-related fatalities worldwide. Matrix metalloproteinases (*MMPs*) are key mediators of tissue remodeling and are linked to tumor aggressiveness, yet there is still a lack of information on the link between genetic changes in *MMPs-1,3* and the onset and progression of OC in Egyptian women. This study examines the effects of immunoreactive biomolecule variations of *MMPs-1,3*, as well as the *MMP-1 (1607 1G/2G)* and *MMP-3 (-1171 5A/6A)* genetic variants, on OC risk and progression in Egyptian women. **Methods**: Tissue specimens embedded in paraffin from 100 OC patients and 60 controls were stained using immunohistochemistry to examine expression of *MMPs-1,3*. *MMP* levels were quantified using ELISA, and single-nucleotide polymorphisms (SNPs) of *MMPs-1,3* were genotyped using polymerase chain reaction–restriction fragment length polymorphism (PCR-RFLP). **Results**: Increased levels of *MMPs-1,3* in OC patients relative to controls, with more of an increase in the late stages (III and IV) than in the early OC stages (I and II). Additionally, the *MMP-1 2G/2G* and *MMP-3 6A/6A* genotypes were more prevalent in OC patients than in controls. Ovarian *MMPs-1,3* were comparatively elevated in the identified genotypes compared to the *1G/1G* and *5A/5A* genotypes, respectively. The transcriptional activity of *MMPs-1,3* showed strong potential for distinguishing patients with epithelial ovarian carcinoma (EOC) from controls, boasting an area under the curve (AUC) of 0.956 and 0.816, respectively. Sensitivity and specificity were 94.0% and 90.0% for *MMP-1* and 80.0% and 73.3% for *MMP-3*, respectively. **Conclusions**: The *MMP-1 2G/2G* and *MMP-3 6A/6A* genotypes are correlated with elevated *MMP-1* and *MMP-3* levels and immunohistochemical expression in carcinomatous ovarian tissues, particularly in advanced stages of OC. This indicates that genetic variations of *MMPs-1,3* could be valuable diagnostic and prognostic markers for OC in Egyptian women. Our findings may carry clinical relevance for optimizing OC therapeutic effectiveness, contribute to the growing body of knowledge on the role of *MMPs*, and shed new light on the genetic background of OC. Future studies with larger sample sizes and comprehensive *MMP* genetic profiling are needed for results validation.

## 1. Introduction

Ovarian carcinoma (OC) is the foremost factor in female carcinoma-related fatalities worldwide, primarily due to late-stage diagnosis resulting from nonspecific and subtle symptoms [1]. In the United States alone, about 22,240 new OC cases and around 14,030 related deaths are annually recorded [2]. Despite extensive research on OC early detection and treatment [3], an effective screening strategy and early diagnostic techniques are essential for improving survival rates and reducing mortality [4,5]. Genetic alterations, including single-nucleotide polymorphisms (SNPs), are implicated in carcinogenesis development and carcinoma progression [6]. Among these, matrix metalloproteinases (*MMPs*) constitute more than 20 proteolytic enzymes with similar structural and functional properties [7]. These zinc-dependent enzymes can be inhibited by extracellular metalloproteinase inhibitors and are classified into four groups based on function: gelatinases, collagenases, membrane-type MMPs (MT-MMPs), and stromelysins [8]. *MMPs* are pivotal in extracellular matrix (ECM) and basement membrane component degradation, processes essential for carcinogenesis, tumor cell growth, and metastasis [9]. Elevated serum and ovarian tissue levels of *MMPs* have been observed in OC patients [10,11]. Genetic polymorphisms in *MMPs* can lead to their overexpression, potentially enhancing tumor aggressiveness and metastasis [12]. Several studies have revealed the correlation between *MMP* genetic alterations and OC risk [13,14]. Specifically, the impact of *MMP-1* promoter SNPs on OC development has been examined [15,16,17]. *MMP-3* is a key regulator of ECM remodeling and an activator of other MMPs [18]. Elevated *MMP-3* expression has been reported in hen OC models and in various human carcinomas [18]. Moreover, *MMP-3* overexpression in OC has been linked to miRNA-200 suppression, which disrupts its role in limiting OC invasiveness [19]. According to these findings and the discrepancies of the previous studies, it is crucial to investigate the association between *MMP* SNPs and OC risk, invasiveness, and metastasis. To our knowledge, limited data exist regarding the relationship between the transcriptional activity and genetic alterations of *MMPs-1,3* and OC susceptibility and progression in Egyptian women. Therefore, this study aims to analyze the correlation of *MMP-1 (-1607 1G/2G)* and *MMP-3 (-1171 5A/6A)* promoter variants with their expression patterns in ovarian tissues, and their potential role as diagnostic and prognostic biomarkers of OC in Egyptian women.

## 2. Materials and Methods

### 2.1. Study Framework

This investigation employed a case–control framework which examined and genotyped 160 subjects, with samples obtained from the Pathology Department Database, Faculty of Medicine, Zagazig University, and Zagazig University Hospitals, Egypt.

### 2.2. Sample Size Calculation

Using the G*Power tool, the required sample size was estimated considering a 0.05 significance level and 80% statistical power, with a 0.3 medium-sized effect. A total of 160 individuals were included: 60 OC-free samples as controls and 100 confirmed OC samples.

The control group comprised 60 individuals with an average age of 45.5 ± 11.1 years (23.5–61 years). These subjects underwent salpingo-oophorectomy for non-malignant conditions, with OC explicitly excluded. Processed ovarian sections were stained using the standard hematoxylin and eosin (H&E) technique to confirm the absence of malignancy before DNA extraction.

The patient group included paraffin-embedded tumor sections from 100 individuals diagnosed with epithelial ovarian carcinoma (EOC) confirmed by histological examination and DNA extraction. The patients’ ages ranged from 23.5 to 82.5 years, with a mean of 47 years (±10.7). Before surgery, none had undergone chemotherapy or radiotherapy. Two independent pathologists evaluated all tissue specimens for further verification. The International Federation of Gynecology and Obstetrics (FIGO) guidelines were used to define the stages and subtypes of EOC [20].

Among the 100 EOC patients, the histological subtypes were distributed as follows: 45 (45%) had serous carcinoma (SC), 15 (15%) had mucinous carcinoma (MC), 27 (27%) had endometrioid carcinoma (EC), and 13 (13%) had clear-cell carcinoma (CC). The histological subtypes were independently verified by pathologists. Cases involving germ cell neoplasm and stromal and sex cord-derived lesions, as well as secondary ovarian involvement from extragonadal primaries, were not included in the analysis.

### 2.3. Molecular and Immunohistochemical Profiling

Tissue values of *MMPs-1,3* were quantified using enzyme-linked immunosorbent assay (ELISA), while immunohistochemical staining was utilized to assess *MMP-1* and *MMP-3* transcriptional intensity. To investigate genetic variations, DNA was isolated and analyzed for SNPs in *MMP-1 (-1607 1G/2G)* and *MMP-3 (-1171 5A/6A)*.

#### 2.3.1. Quantification of *MMPs-1,3* Within Ovarian Tissue

The levels of *MMPs-1,3* in ovarian tissue were quantified using a sandwich enzyme-linked immunosorbent assay (ELISA) [R&D Systems Minneapolis, MN, USA; Catalog #DMP 300]. Samples (1 mm) were excised from paraffin-embedded blocks using a sterile surgical blade. For each sample (25–100 mg), homogenization was performed using a cell lysis buffer. The homogenate was subsequently placed in a microtube and subjected to centrifugation. After collection, the supernatant was employed for quantification of *MMPs-1,3* using ELISA.

#### 2.3.2. DNA Extraction and Genotyping

Following the deparaffinization of ovarian tissue samples, the phenol/chloroform method with proteinase K digestion was employed for genomic DNA isolation according to previously established protocols [21]. The sample was treated with Tris-saturated phenol (pH 8) and spun at 12,000 rpm for 3 min. Afterward, 300 µL of a 1:1 phenol/chloroform mixture was introduced to the supernatant, and DNA precipitation was induced by the addition of 2.5 volumes of ethanol. After rinsing with 70% ethanol, the DNA pellet was dried and rehydrated in 70 µL distilled water for subsequent PCR analysis.

#### 2.3.3. Selection Criteria for *MMP-1 (-1607, 1G/2G)* and *MMP-3 (-1171, 5A/6A)* Polymorphisms and Genotyping

##### Selection Criteria for Polymorphisms

The promoter polymorphisms *MMP-1 (-1607 1G/2G*, *rs1799750*) and *MMP-3 (-1171 5A/6A*, *rs3025058*) were selected for analysis based on their functional impact on gene transcription, their involvement in ECM remodeling, and their previously reported associations with tumorigenesis and metastatic potential in various malignancies [13,14].

The *MMP-1 (-1607 1G/2G*) polymorphism involves the insertion of a guanine nucleotide, creating a *2G* allele that introduces an Ets transcription factor binding site, thereby enhancing the promoter activity and resulting in elevated *MMP-1* expression. Increased *MMP-1* expression has been associated with enhanced degradation of interstitial collagens (types I and III), facilitating tumor invasion and metastasis [15,16,17]. Similarly, the *MMP-3 (-1171 5A/6A)* variant is characterized by an adenine insertion/deletion polymorphism within the promoter region, where the *5A* allele exhibits greater transcriptional activity than the *6A* allele due to reduced affinity for a repressor nuclear protein [19]. *MMP-3* not only degrades multiple ECM components directly but also activates other MMPs, such as MMP-1, amplifying the proteolytic cascade involved in tumor progression [18,19].

These SNPs were chosen because they represent functionally relevant promoter variants with regulatory roles in gene expression, and have been implicated in the susceptibility and progression of breast, lung, gastric, and colorectal cancers [8,9]. However, their contribution to ovarian cancer, particularly in Middle Eastern and North African populations, remains inadequately explored. Their selection was thus based on their functional significance, their prior cancer association, and the existing gap in population-specific data regarding ovarian cancer risk and prognosis.

Genotyping of *MMPs-1,3* SNPs was performed via the polymerase chain reaction–restriction fragment length polymorphism (PCR/RFLP) technique [22,23].

***MMP-1* SNP Primers**:

Forward primer: 5′-TGACTTTTAAAACATAGTCTATGTTCA-3′.
Reverse primer: 5′-TCTTGGATTGATTTGAGATAAGTCATAGC-3′.

***MMP-3* SNP Primers**:

Sense primer: 5′-GGTTCTCCATTCCTTTGATGGGGGGAAAGA-3′.
Antisense primer: 5′-CTTCCTGGAATTCACATCACTGCCACCACT-3′.

A 25 µL PCR was prepared with 100 ng of template DNA and 2.5 µL of 10 × buffer, 2.0 mM MgCl_2_, 0.2 mM of each dNTP (Sigma Chemical Co., St. Louis, MO, USA), 0.2 mM of forward and reverse primers (Biosynthesis), and Taq DNA polymerase (2.5 units) (Hoffman-La Roche, Branchburg, NJ, USA). Reactions were performed on a Perkin Elmer 4800 thermal cycler (PTC-100, MJ Research, Watertown, MA, USA). The thermal profile began with denaturation at 95 °C for 2 min, followed by 35 cycles of 94 °C for 45 s, 50 °C for 60 s (for *MMP-1*) or 63 °C for 90 s (for *MMP-3*), and 72 °C for 60 s. The reaction concluded with a 10 min extension at 72 °C.

**Figure 1:**
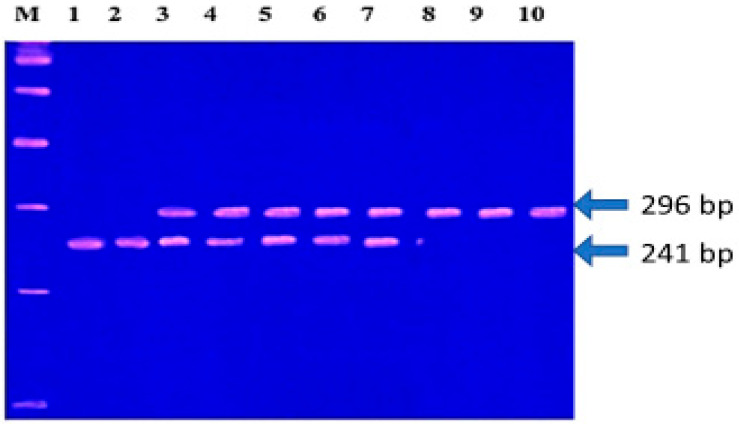
*MMP-1 (-1607, 1G/2G)* gel electrophoresis bands: M = 100 bp marker; lines 1 and 2 for *1G/1G*; lines 3–7 for *1G/2G*; lines 8, 9, and 10 for *2G/2G*.

**Figure 2:**
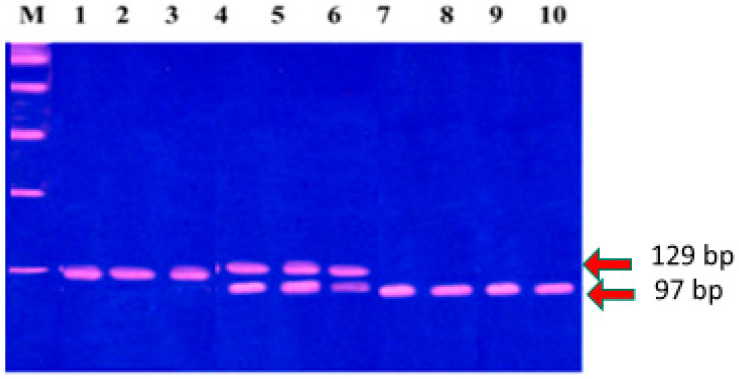
*MMP-3 (-1171, 5A/6A)* gel electrophoresis bands: line M = 100 bp marker; lines 1–3 for *6A/6A*; lines 4–6 for *5A/6A*; lines 7–9 and 10 for *5A/5A*.

SNP genotyping was conducted by enzymatic digestion of the reaction products. *MMP-1* was analyzed using 10 U of Alu I at 37 °C for 16 h, while 5 U of Nsp I restriction enzyme was used at 65 °C for 4 h to analyze *MMP-3* SNPs. The resulting digested products were resolved using 3% polyacrylamide gel electrophoresis with ethidium bromide (5 mg/mL) staining. DNA fragments were visualized under UV light, and their sizes were determined using a 100 base pair (bp) marker. For *MMP-1*, *the 2G/2G* genotype exhibited a single band of 269 bp; the *1G/1G* genotype displayed 241 and 28 bp bands; and the *1G/2G* genotype showed fragments of 269, 241, and 28 bp (Figure 1). For *MMP-3*, the *5A/5A* genotype was characterized by 97 and 32 bp bands, while the *6A/6A* genotype was identified by a 129 bp fragment. The *5A/6A* genotype produced fragments of 129, 97, and 32 bp (Figure 2).

Immunohistochemical staining was carried out on 4 μm tissue sections using mouse monoclonal antibodies against human *MMP-1* (1:100, Labvision) and *MMP-3* (1:50, Novus Biologicals). The avidin–biotin–immunoperoxidase method was utilized for the staining process. The tissue sections were placed at 60 °C for approximately 16 h, before undergoing xylene-based deparaffinization and rehydration through a series of graded alcohol solutions. A 10 min incubation in 0.3% hydrogen peroxide/methanol was used to neutralize intrinsic peroxidase activity on the tissue sections. To retrieve masked antigens, the sections were heated at 100 °C for 15 min in Dako’s Target Retrieval Solution. Immunostaining was performed using the Vectastain Elite ABC-Peroxidase system in accordance with the supplier’s guidelines. After every incubation, phosphate-buffered saline (PBS) was used to wash the slides. Antibody detection was accomplished by incubating the sections with Diaminobenzidine (DAB) [Sigma FAST™ DAB, Sigma-Aldrich, St. Louis, MO, USA]. Nuclear counterstaining was performed using hematoxylin. The sections were then dehydrated before the examination.

#### 2.3.4. *MMP-1* Immunohistochemical Analysis

**Figure 3:**
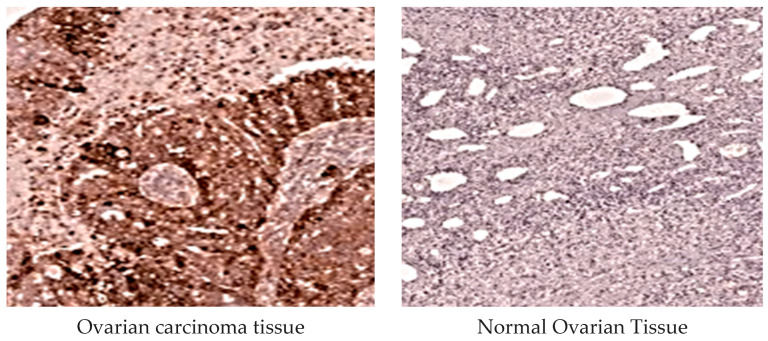
Comparison of *MMP-1* protein levels between epithelial ovarian carcinoma and normal tissues. Protein immunoreactivity of the tumor cells’ cytoplasm showed more expression of *MMP-1* than normal ovarian tissues and appeared in more antibody staining of the tumor ovarian tissue than normal.

*MMP-1* protein expression was evaluated based on the cytoplasmic immunoreactivity in tumor cells. Specimens were classified with a positive designation for stained specimens and a negative designation for those without staining. A scoring system ranging from 0 to 3 was used to assess tumor samples according to the extent of the staining. Tumors were deemed positive if the proportion of stained cells exceeded 1%. The intensity of staining was categorized as weak (+), moderate (++), or strong (+++) based on the percentage of positive tumor cells: weak (+) for 1–25%, moderate (++) for 25–50%, and strong (+++) for >50% of the cells (Figure 3).

#### 2.3.5. *MMP-3* Immunohistochemical Examination

**Figure 4:**
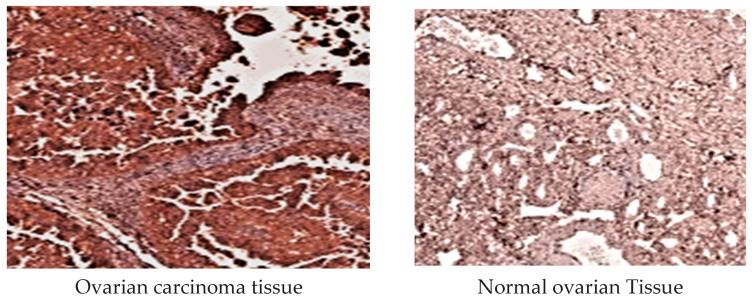
Comparison of *MMP-3* protein levels between epithelial ovarian carcinoma and normal tissues. Protein immunoreactivity of the tumor cells’ cytoplasm showed more expression of *MMP-3* than in normal ovarian tissues and appeared with more antibody staining of the tumor ovarian tissue than normal.

*MMP-3* immunoreactivity was assessed in the cytoplasm of tumor cells. Samples were classified as negative or positive based on the presence of staining. Tumors were marked as positive if >1% of malignant cells exhibited staining. Intensity was graded as weak, moderate, or intense, with >50% positive staining categorized as intense. Specimens were scored on a scale of 0–2 according to staining intensity (Figure 4).

### 2.4. Statistical Analysis

Data analysis was performed using software (16.0) (SPSS Inc., Chicago, IL, USA). Ovarian tissue levels of *MMPs-1,3* were expressed as mean ± standard deviation (SD). A one-way ANOVA test (F-test) was applied as it evaluates the variance within and between groups. The F-test was used to determine if the observed differences were statistically significant, thereby providing insight into how EOC stages, histological subtypes, and genetic variations might have influenced the studied variables. A *t*-test was used for pairwise comparisons. The Hardy–Weinberg equilibrium of the genotypes of *MMPs-1,3* was examined by the chi-square (χ^2^) test.

Odds ratios (ORs) and 95% confidence intervals (CIs) were computed to assess the association between *MMP-1* and *MMP-3* alleles and the risk of OC at various stages of the disease. ROC curve analysis was utilized to determine the optimal sensitivity and specificity, with statistical significance defined at *p* < 0.05.

### 2.5. Ethical Approval and Informed Consent

Ethical approval for conducting this study was secured by the Institutional Review Board (IRB) of the Faculty of Medicine’s Research Ethics Committee at Zagazig University (IRB no. #9762/5-2021, dated 22 May 2021). The samples were retrieved from the Pathology Department Database of Zagazig University Faculty of Medicine and Zagazig University Hospitals. To ensure the participants’ privacy, stringent confidentiality protocols were employed, and the dataset was used exclusively for research purposes. Identifiable personal information, including names and addresses, was omitted to maintain participant anonymity. The research adhered completely to ethical principles and guidelines, including the Declaration of Helsinki, which outlines the ethical considerations for medical research involving human subjects.

## 3. Results

Paraffin-embedded tumor tissue samples from 160 subjects were examined, including 60 control subjects with an average age of 45.64 ± 11.1 years (23.5–61 years) and 100 patients with histologically diagnosed EOC, whose average age was 47 ± 10.7 years (23.5–82.5 years).

**Figure 5:**
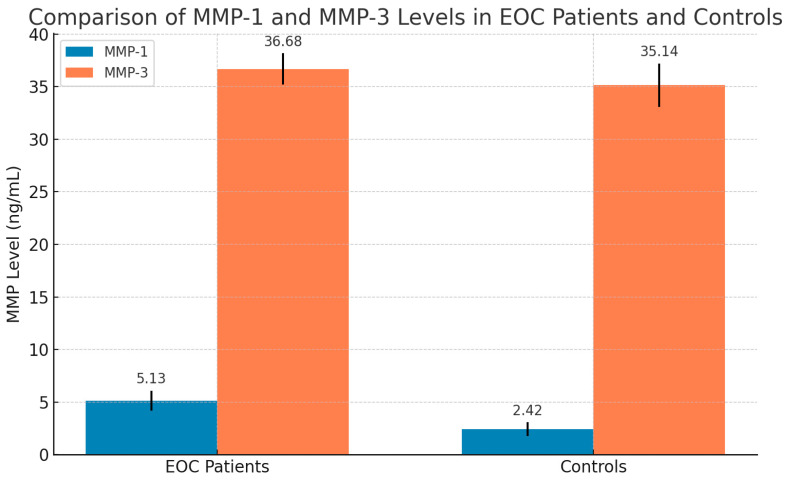
Comparison of the mean ± S.D of *MMP-1* and *MMP-3* between controls and epithelial ovarian carcinoma patients (EOC), as determined by *t*-test and *p*-value. Bars represent mean values. *MMP-1* levels were significantly higher in EOC patients compared to controls (t = 23.08, *p* < 0.001). *MMP-3* levels were elevated in EOC patients compared to controls (t = 3.55, *p* = 0.001).

Age-related discrepancies between the patient and control groups were not statistically meaningful. ELISA was used to measure the levels of *MMPs-1,3* in ovarian tissue, examining the influence of SNPs on their expression. The results revealed significantly elevated levels of *MMPs-1,3* in EOC patients relative to controls. The mean *MMP-1* level was 5.13 ± 0.96 ng/mL in EOC patients versus 2.42 ± 0.66 ng/mL in controls (t = 23.08, *p* = 0.000). The mean *MMP-3* level was 36.68 ± 1.5 ng/mL in patients and 35.14 ± 2.06 ng/mL in controls (t = 3.55, *p* = 0.001) (Figure 5).

Table 1 presents the distribution of clinical stages and histological subtypes among the epithelial ovarian carcinoma patients included in the study (*n* = 100). The majority of patients were diagnosed at Stage II (43%), followed by Stage I (30%), indicating a relatively early-stage presentation in this cohort. Regarding histological classification, serous carcinoma was the most prevalent subtype (45%), consistent with global epidemiological trends. Endometrioid (27%), mucinous (15%), and clear cell carcinomas (13%) were also represented, reflecting the typical heterogeneity of epithelial ovarian cancer.

Table 2 represents the *MMP-1* and *MMP-3* levels according to EOC stage and histological subtype. MMP-1 and MMP-3 exhibited enhanced levels with the progression of EOC stages (F = 21.14, *p* = 0.000; F = 7.23, *p* = 0.000, respectively). Specifically, an increase in *MMP-1* levels was detected in advanced stages (III and IV) relative to the early stage (I) (t = 3.543, *p* = 0.011; t = 4.338, *p* = 0.004). Moreover, *MMP-3* concentrations were increased more in stage IV than in stage I (t = 4.025, *p* = 0.001). Concentrations of *MMPs-1,3* remained consistent across different histological types of EOC (F = 0.749, *p* = 0.32; F = 1.355, *p* = 0.261).

To investigate the impact of *MMP-1 (-1607, 1G/2G)* and *MMP-3 (-1171, 5A/6A)* SNPs on EOC development and prognosis, DNA from 60 normal ovarian tissues and 100 EOC tissue samples was analyzed using PCR-RFLP. The resulting PCR-RFLP fragments are shown in Figure 1 and Figure 2.

Next, the link between *MMP-1 (-1607)* and *MMP-3 (-1171)* SNPs and EOC was analyzed. Table 3 illustrates the distribution of MMP-3 *(-1171; 5A/6A)* and MMP-1 *(-1607; 1G/2G)* genotypes and allelic frequencies across different stages of EOC. For *MMP-3*, the heterozygous *5A/6A* genotype was the most frequent overall, while the homozygous *6A/6A* genotype showed a progressive increase with advancing tumor stage, reaching 50% in Stage 4 patients. Although the genotype distribution was statistically significant (χ^2^ = 20.97, *p* = 0.002), the allele distribution showed a borderline association with tumor stage (χ^2^ = 7.38, *p* = 0.061). In the case of *MMP-1*, a clear shift was observed toward the homozygous *2G/2G* genotype in higher stages, particularly Stage IV (50%), with a corresponding increase in *2G* allele frequency. Both the genotype (χ^2^ = 29.067, *p* < 0.001) and allele (χ^2^ = 12.83, *p* = 0.005) distributions demonstrated statistically significant associations with tumor progression, suggesting a potential role of the MMP-1 promoter polymorphism in disease advancement. 

Hardy–Weinberg equilibrium was observed for both *MMP-1* and *MMP-3* genotypes. The genotype distributions and allele frequencies of *MMP-1* and *MMP-3* SNPs between normal ovarian and EOC tissues were compared using the chi-square (χ^2^) test (Table 4). The *MMP-1 (-1607) 2G/2G* genotype and the 2G allele frequency were significantly elevated in EOC patients compared to in controls (χ^2^ = 31.539, *p* = 0.000). The odds ratio for the 2G allele was 0.23 (95% CI: 0.136–0.401; χ^2^ = 30.10, *p* = 0.000). Similarly, the *MMP-3 (-1171) 5A/6A* genotype and 6A allele frequencies were significantly enhanced in EOC patients (χ^2^ = 16.626, *p* = 0.000; χ^2^ = 16.370, *p* = 0.000; OR = 0.355, 95% CI: 0.213–0.505). No statistically significant link was found between *MMP-1* and *MMP-3* SNPs and EOC histological subtypes (χ^2^ = 4.36, *p* = 0.620; χ^2^ = 11.826, *p* = 0.678; χ^2^ = 2.62, *p* = 0.453). However, the *MMP-3 6A* allele was significantly more frequent in the SC subtype (χ^2^ = 18.266, *p* = 0.041) (Table 4).

Additionally, an immunohistochemical analysis of the protein expression of *MMPs-1,3* was performed in 100 EOC and 60 control ovarian tissue samples (Figure 3 and Figure 4). EOC tissues exhibited more intense antibody staining of *MMPs-1,3* than normal ovarian tissues. The immunoreactivity of *MMP-1* and *MMP-3* in tumor cells was significantly higher than in normal tissues. *MMP-1 2G/2G* and *MMP-3 6A/6A* genotype carriers showed more intense antibody staining than *1G/1G* and *5A/5A* genotype carriers.

The F-test assessed the correlation between *MMP-1* and *MMP-3* SNPs and tissue levels. *MMP-1* and *MMP-3* tissue levels were significantly higher in the *2G/2G* and *6A/6A* genotype carriers in both normal and carcinomatous ovarian tissues (Table 5).

### Diagnostic Value of MMP-1 and MMP-3 Alleles

**Figure 6:**
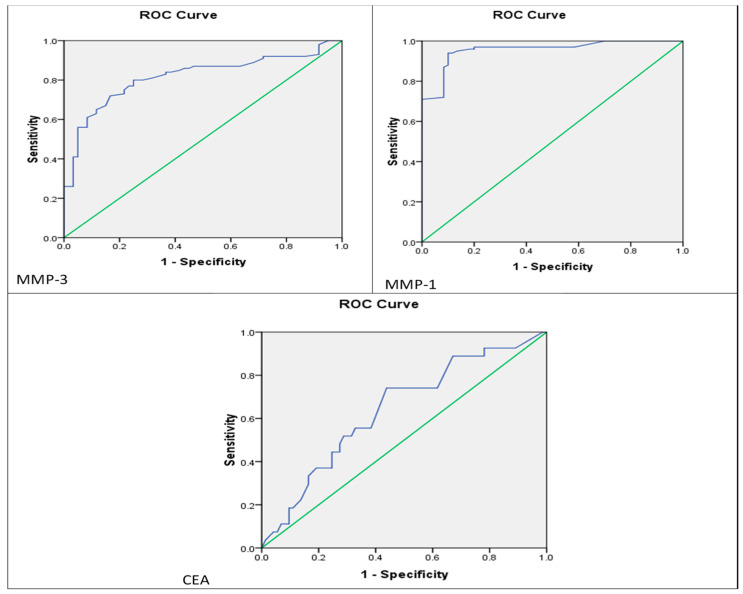
Receiver operating characteristic (ROC) curve for levels of *MMP-1,3* and CEA discriminating between EOC patients and controls; the blue line represents the ROC curve of each biomarker, indicating its diagnostic performance across different thresholds. The green diagonal line denotes the reference line (AUC = 0.5), which reflects a test with no discriminative power. A larger area under the blue curve (AUC) indicates better diagnostic accuracy.

To assess the predictive ability of *MMP-1* and *MMP-3* SNPs, ROC curve analysis was conducted. The area under the ROC curve (AUC) for *MMP-1* was 0.956 (95% CI: 0.926–0.986), while, for *MMP-3*, it was 0.816 (95% CI: 0.749–0.883), effectively differentiating EOC patients from controls. The optimal sensitivity and specificity for *MMP-1* were 94.0% and 90.0%, respectively, while, for *MMP-3*, they were 80.0% and 75.3%. In comparison to the CEA marker (AUC = 0.64, 95% CI: 0.52–0.76), *MMP-1* and *MMP-3* exhibited more diagnostic accuracy. These results indicate that the allelic variants of *MMP-1* and *MMP-3* may serve as more sensitive and specific biomarkers for EOC than CEA (Table 6 and Figure 6).

**Figure 7:**
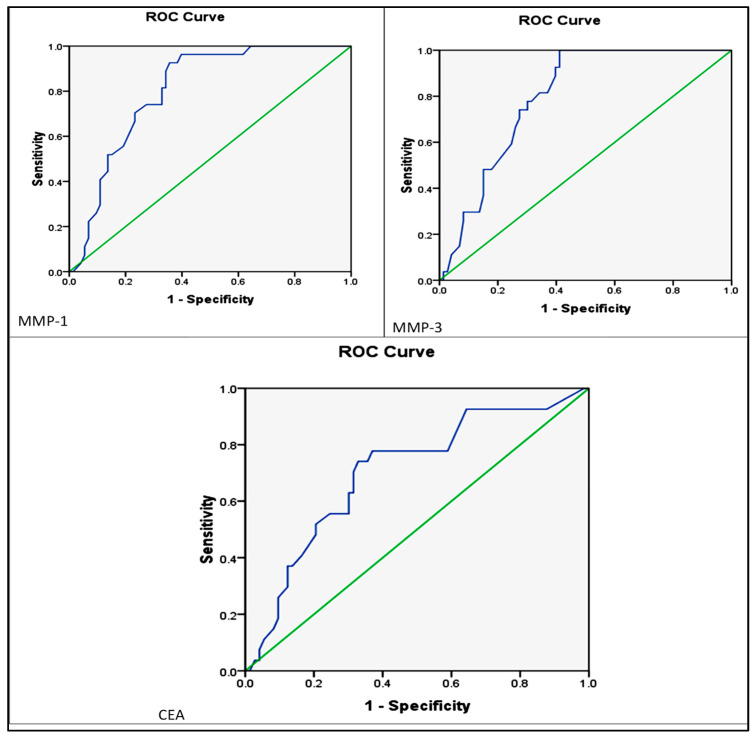
Receiver operating characteristic (ROC) curve for levels of *MMP-1,3* and CEA, differentiation between EOC stages I and II and stages III and IV. The blue line represents the ROC curve for each biomarker, reflecting its diagnostic performance. The green diagonal line represents the line of no discrimination (AUC = 0.5). A greater area under the blue curve (AUC) indicates higher discriminatory ability between early and advanced disease stages.

Additionally, ROC analyses revealed that *MMP-1* (AUC = 0.806, 95% CI: 0.721–0.890) and *MMP-3* (AUC = 0.797, 95% CI: 0.713–0.80) levels had better discriminatory ability between stages I and II and stages III and IV compared to CEA (AUC = 0.703, 95% CI: 0.588–0.819) (Table 7 and Figure 7).

## 4. Discussion

The delayed detection of OC underscores the urgent need for reliable biomarkers to facilitate early diagnosis and serve as prognostic biomarkers for disease progression [1,24]. *MMPs*, a class of enzymes involved in the degradation of the extracellular matrix (ECM), play a key role in the development and dissemination of several carcinomas, primarily OC [25]. Their ability to facilitate tumor cell invasion by breaking down ECM components makes them a critical factor in carcinoma aggressiveness [24,25]. The functional implication of *MMPs-1,3* in tumor progression has been well-documented, suggesting that alterations in their expression or genetic structure could potentially serve as valuable biomarkers in OC [26]. *MMPs-1,3* have been noted in the breakdown of collagen in the ECM, a key step in facilitating the invasion of tumor cells into surrounding tissues. Previous studies have suggested that elevated levels of *MMP-1* correlate with increased tumor aggressiveness and poor prognosis in various types of carcinomas [27,28,29].

Additionally, alterations in the promoter regions of *MMP-1 (-1607 1G/2G)* and *MMP-3 (-1171 5A/6A)* genes have been linked to changes in their expression levels, which, in turn, are associated with a poorer prognosis in OC [28]. This suggests that genetic alterations in *MMP-1* and *MMP-3* could function as a diagnostic/prognostic indicator for identifying patients at higher risk for aggressive disease. While there is a strong rationale for studying *MMPs* in OC, there is still a lack of comprehensive research concerning the influence of the levels of *MMPs-1,3* and their genetic variants on the progression of OC, especially in Egyptian women. While *MMP-1* has been studied in other carcinomas, such as endometrial carcinoma, its role in OC in Egypt remains underexplored. Mackawy et al. [30] demonstrated the involvement of *MMP-1* in endometrial carcinoma risk and aggressiveness among Egyptian women [30], yet similar research for OC is lacking. This highlights the need for further studies to understand the specific impact of *MMP-1* and *MMP-3* on OC progression. Given the genetic diversity across populations, it is crucial to explore how genetic changes in *MMPs* contribute to the risk of OC in Egyptian women.

We designed this research to bridge the existing knowledge gap by analyzing the immunoreactive protein levels of *MMPs-1,3*, along with their respective genetic polymorphisms (*MMP-1 -1607 1G/2G* and *MMP-3 -1171 5A/6A*), in OC patients. Understanding the correlation between these biomarkers and the disease’s progression could provide valuable insights into potential diagnostic tools and targeted therapies for OC in Egypt. Moreover, by identifying specific genetic alterations that are more prevalent in this population, we can better tailor prevention and treatment strategies to improve patient outcomes.

In this study, we examined 100 Egyptian patients with histologically confirmed EOC. The majority of our EOC patients were diagnosed with stage II (43%), and 45% were identified as having the SC histological subtype. We focused on examining the relationship between the genetic polymorphisms of *MMPs-1,3* and the OC development risk, as well as evaluating their association with EOC stages and histological subtypes, to assess their potential as diagnostic and prognostic biomarkers. Our findings revealed that the protein expression levels of *MMPs-1,3* were elevated in EOC tissue compared to normal ovarian tissue, with a remarkable increase observed in the late stages of EOC compared to the early stages. Nonetheless, the analysis revealed non-significant variation in the levels of *MMPs-1,3* across the EOC histological subtypes. Based on these findings, the levels of *MMPs-1,3* seem to reflect disease progression through different stages, and they appear to be less affected by the specific histological subtypes of EOC. ROC curve analysis indicated that *MMPs-1,3* could be valuable diagnostic biomarkers for distinguishing between EOC patients and controls, with sensitivities of 94.0% and 80.0%, respectively, and specificities of 90.0% and 75.3%. These diagnostic capabilities were superior to the CEA marker, which demonstrated lower sensitivity (55.6%) and specificity (61.6%). These results indicate that the tissue expression of allelic variants of *MMPs-1,3* may offer greater sensitivity and specificity as a diagnostic biomarker for EOC compared to CEA. These results align with those of Lin et al. [31], who found that *MMP-9* and *MMP-3* could initiate epithelial-to-mesenchymal transition (EMT) by disrupting E-cadherin and reducing the expression of epithelial markers, a critical early event in OC development. Similarly, *MMP-1* and *MMP-3* are engaged in promoting EMT, a process that facilitates tumorigenesis and accelerates the invasion of tumor cells [32]. These findings reinforce the potential of *MMPs*, particularly *MMPs-1,3*, as key drivers of carcinoma progression and as promising diagnostic biomarkers in EOC.

*MMP-3* and *MMP-9* have been shown to have similar impacts on EMT and MT1-MMP expression in active metastatic OC cells [33]. Elevated expression of MT1-MMP is linked to increased formation of migratory cell aggregates (MCAs) and enhanced disseminative affinity [34]. MCAs have been implicated in higher invasive and adhesive capabilities compared to normal cells [35]. These findings support the role of *MMPs* in promoting carcinoma cell dissemination and invasion.

In agreement with our data, Sun et al. [19] demonstrated that the excessive expression of *MMP-3* in ovarian carcinomatous tissue is associated with the downregulation of miRNA-200, leading to a reduced ability of miR-200 to repress OC aggressiveness and metastasis. This indicates that *MMP-3* may enhance the metastatic capacity of OC by influencing the expression of critical regulatory microRNAs. Carey et al. [36] reported that *MMP* activity is fundamentally involved in both the initiation and advancement of ovarian carcinogenesis, from tumor initiation to dissemination. *MMPs*, in combination with Snail transcription factors, form a regulatory transcriptional feedback loop that enhances the spread and dissemination of tumor cells [37,38]. Our findings align with this perspective, suggesting that *MMPs* may be integral to the metastatic process in OC.

Our results are consistent with those of Behrens et al. [39], Hantke et al. [40], and Stadlmann et al. [41], who documented that *MMP-1* is involved in OC and that its expression patterns correlate with both the stage of the disease and the tumor histological subtype. This further supports the potential of *MMP-1* as a marker for disease progression in OC.

Agarwal et al. [42] identified that *MMP-1* may activate protease-activated receptor-1 (PAR-1), which, in turn, induces the production of various vasculogenic factors from OC tissues, leading to endothelial cell proliferation, tubule formation, and transmigration [43], ultimately promoting EOC cell invasiveness [44]. Similarly, Wang et al. [45] and their team revealed a marked upregulation of *MMP-3* expression in advanced stages of OC [45]. This further emphasizes the role of *MMPs* in the advanced stages of OC, where they may facilitate tumor progression and metastasis.

Contrary to our findings, Karabulut et al. [46] reported that PAR1 and *MMP-1* blood levels did not work as predictive or prognostic indicators in patients with EOC. Such a discrepancy could stem from variations in the demographic or clinical characteristics of the study cohorts, differing methodologies, or the biological variability in the levels of these markers between different cohorts. However, this study underscores the potential of the genetic variants of *MMPs-1,3* as prognostic markers for EOC. To examine the impact of *MMP-1 (-1607 1G/2G)* and *MMP-3 (-1171 5A/6A*) genetic variants on the risk and severity of EOC, we assessed the frequency of the *MMP-1 (1G/2G)* and *MMP-3 (5A/6A)* genotypes and alleles in both EOC and normal ovarian tissue. We also investigated their correlation with EOC stages and histological subtypes through ROC curve analysis to determine their prognostic value. Our results demonstrated that the *MMP-1 (-1607 2G/2G)* and *MMP-3 (-1171 6A/6A)* genotypes were more frequent in EOC patients compared to controls, indicating their association with increased risk of EOC development. Furthermore, according to the FIGO classification, the *MMP-1 (-1607 1G/2G)* and *MMP-3 (-1171 5A/6A)* genotypes were more prevalent in late-stage EOC compared to the early stages. While the *MMP-3 (-1171 6A)* allele exhibited a higher frequency in advanced stages of EOC, the increase was not statistically validated, likely due to the limited cohort. Moreover, the *MMP-3 6A* allele was significantly more prevalent in the SC subtype of EOC, highlighting a potential subtype-specific association.

To investigate the potential correlation between *MMP-1 (1G/2G)* and *MMP-3 (5A/6A*) SNPs and the protein expression of *MMP-1,3* in carcinoma cells, an immunohistochemical analysis of these proteins was executed in both malignant and normal ovarian tissues. The results showed that the *MMP-1 2G/2G* and *MMP-3 6A/6A* genotype carriers had more frequent staining with antibodies than *1G/1G* and *5A/5A*. Elevated levels of *MMPs-1,3* were particularly notable in late-stage EOC, suggesting that these genotypes could serve as indicators of a poor prognosis for Egyptian patients with OC. Additionally, our data revealed that *MMPs-1,3* could function as prognostic markers, with sensitivities of 74.1% and 77.8%, and specificities of 72.6% and 70.0%, respectively, for distinguishing between early and advanced stages of EOC.

Consistent with our results, Kanamori et al. [12] highlighted the *MMP-1 2G* allele at *rs1799750* as a possible genetic risk factor for OC among Japanese women. This supports the idea that *MMP-1* genetic variants, particularly the *2G* allele, may be relevant to OC susceptibility across different populations. Increased *MMP-1* expression levels have been correlated with high mortality rates in several malignancies, including breast, lung, gastric, and colon carcinomas [47]. The *MMP-1 [1G/2G]* SNP may influence the regulation of *MMP-1* expression and thus affect OC progression and patient survival [47]. These findings highlight the potential of *MMP-1* and *MMP-3* as diagnostic and prognostic biomarkers of EOC.

In our cohort, while the *6A* allele frequency was higher in late-stage patients, *MMP-3* protein expression was paradoxically elevated in cases with the *MMP-3 6A* allele.

In contrast, Ye et al. [48] demonstrated that the *MMP-3 (-1171, 5A/6A*) promoter alteration is linked with the progression of atherosclerosis, shedding light on how this mutation could impact *MMP-3* expression. However, they demonstrated that the *6A* allele is linked to lower promoter activity and reduced MMP-3 expression compared to the *5A.* Similarly, Bondi et al. [49] also reported that the *5A/5A* and *5A/6A* genotypes exhibited enhanced promoter activity relative to the *6A* allele in carcinoma patients compared to healthy individuals. Their results indicate that the *5A* polymorphism in the *MMP-3* promoter may contribute to tumor formation and advancement.

This suggests that additional regulatory factors or environmental factors may modulate *MMP-3* expression in OC beyond the promoter polymorphism alone. For example, post-transcriptional regulation, tumor microenvironment cues, or linkage disequilibrium with other functional variants could contribute to elevated *MMP-3* levels despite the presence of the lower-activity *6A* allele. The complex interaction between genotype and phenotype in cancer progression necessitates further functional studies to dissect the precise molecular mechanisms governing *MMP-3* expression and activity in ovarian cancer.

Furthermore, the *5A/6A* mutation in the *MMP-3* promoter locus leads to elevated levels of *MMP-3* transcription and its localized expression [50]. Ghilardi et al. [50] also found that this mutation might serve as a marker for poor prognosis and worse outcomes in breast carcinoma, linking it to enhanced carcinoma invasiveness.

*MMP-3 (5A/6A)*, linked with the *MMP-1 1G/2G* variant, results in Glu-to-Lys (*G* to *A*) amino acid exchange in the *MMP-3* propeptide region. This region is cleaved to produce the active form of the enzyme. This modification could alter the activation process of *MMP-3* and its subsequent cleavage, which has several protease cleavage sites within the catalytic region [51].

A recent meta-analysis conducted by Abdul Aziz et al. [52] explored the correlation of the *MMP-3 (-1171 5A/6A)* SNP with various types of carcinoma. The analysis found a significant link between this polymorphism and increased susceptibility to esophageal, colorectal, gastrointestinal, and breast carcinomas, with a 1.56-fold increase for gynecological carcinomas and a 2.40-fold increase for hepatocellular carcinoma.

Opposing findings from some studies showed no significant discrepancies in the *MMP-3 5A/6A* genotypes between individuals with OC and normal subjects. They concluded that genetic mutations in *MMP-1* and *MMP-3* might not be implicated in OC development and are not associated with the risk of OC [14,53]. Their subgroup assessment, considering the ethnicity of the participants, showed comparable results. Wang et al. [26] conducted a meta-analysis involving five studies with 754 patients and 1184 controls, exploring the connection between the *MMP-1 (1G/2G)* SNP and OC. Their analysis revealed a non-significant relationship between this mutation and OC development. Additionally, Ju et al. [15] did not observe any link between this SNP and OC in Korean women.

Genome-wide association studies (GWAS) have not identified significant associations between the *MMP-1 (-1607 1G/2G, rs1799750*) and *MMP-3 (-1171 5A/6A, rs3025058)* promoter polymorphisms and OC risk. These specific variants have not emerged as genome-wide significant loci in large-scale GWAS of OC. However, a meta-analysis by Zhu and Sun [14] comprehensively evaluated the association between *MMP-1 (rs1799750)* and *MMP-3 (rs3025058)* promoter polymorphisms and OC risk. The study concluded that neither polymorphism was significantly associated with OC susceptibility across various genetic models, including homozygote, heterozygote, dominant, recessive, and additive models. Subgroup analyses by ethnicity also revealed no significant associations [14]. These findings suggest that, based on current evidence, these specific *MMP* polymorphisms may not be major contributors to OC susceptibility. It is important to note that, while GWAS provide valuable insights into genetic risk factors for diseases, they may not detect all relevant variants, especially those with small effect sizes or those that are rare in the population. Therefore, further studies, particularly in diverse populations, may be necessary to fully elucidate the role of these polymorphisms in OC risk.

The discrepancies observed between the research findings could be explained by differences in sample size, population ethnicity, and study methodologies. Larger and more rigorous studies are essential to provide a clearer understanding of the relationship between *MMP* genetic variants and OC risk, as well as to assess their correlation with disease progression in Egyptian women.

Our study contributes novel insights in several key aspects:

- Population-specific focus: Unlike most previous studies, which were conducted in Asian or Caucasian populations, our study is among the first to investigate *MMP-1 (-1607 1G/2G)* and *MMP-3 (-1171 5A/6A)* polymorphisms specifically in Egyptian women with EOC, highlighting potential ethnicity-specific risk factors.
- Combined analysis of genotype and protein expression: In addition to genotyping, we performed an immunohistochemical analysis of *MMP-1* and *MMP-3* protein levels, demonstrating a correlation between certain genotypes *(2G/2G and 6A/6A)* and elevated protein expression in EOC tissues, particularly in late-stage disease. This integrative approach provides a more functional and prognostically relevant perspective than previous genetic-only studies.
- Diagnostic and prognostic value: Our ROC analysis revealed that *MMPs -1,3* protein levels exhibited higher sensitivity and specificity compared to CEA, suggesting their superior potential as diagnostic and prognostic biomarkers for EOC.

These novel findings enhance our understanding of *MMP* polymorphisms in OC within a North African population and underscore their potential clinical relevance in early detection and prognosis.

### Limitations and Recommendations

The modest sample size may have compromised the statistical power and generalizability of the findings. Larger, multicenter studies with a more diverse population are needed to confirm these results. While we investigated the connection between *MMPs-1,3* and tumor stage and histological subtype, we did not assess their relationship with patient survival outcomes or treatment response, which could provide further prognostic insights. Additionally, environmental and lifestyle factors that may influence *MMP* expression and OC progression were not considered in this study. Longitudinal studies are warranted to evaluate the prognostic impact of the genetic variants of *MMPs-1,3* on patient survival and treatment response. Functional studies are also recommended to investigate the molecular mechanisms underlying the role of these genetic polymorphisms in OC. Moreover, integrating expression analysis of *MMPs-1,3* with other biomarkers may enhance the accuracy of OC diagnosis and prognosis. The application of targeted therapies aimed at modulating MMP activity could also be a promising avenue for improving treatment outcomes in OC patients.

## 5. Conclusions

To our knowledge, this is one of the few studies to analyze the correlation of *MMP-1 (1G/2G)* and *MMP-3 (5A/6A)* genetic variants and OC risk and progression in Egyptian women. The results concluded that *MMP-1 2G/2G* and *MMP-3 6A/6A* genotypes are linked with elevated levels of *MMPs-1,3*, as well as immunohistochemical expression in carcinomatous ovarian tissues. This association was particularly significant in the advanced stages of EOC according to the FIGO classification. Therefore, these genetic variants could serve as promising diagnostic and prognostic indicators for OC, particularly within an Egyptian cohort. Our findings may have significant clinical implications for improving treatment outcomes and the therapeutic effectiveness of OC. Additionally, they add to our expanding understanding of the involvement of *MMPs* in carcinoma and shed new light on the genetic background of OC.

However, further validation with more diverse populations is needed, and will influence future diagnostic and treatment strategies, ultimately making a meaningful impact on the early identification and effective treatment of OC worldwide.

## Figures and Tables

**Table 1:** Epithelial ovarian carcinoma stages and histological subtype distribution in studied patients (**total *n* = 100**).

Ovarian Carcinoma Stages	Frequency [*n*%]
Stage I	*n* = 30, 30%
Stage II	*n* = 43, 43%
Stage III	*n* = 15, 15%
Stage IV	*n* = 12, 12%
**Histological subtype**	
SC	*n* = 45, 45%
MC	*n* = 15, 15%
EC	*n* = 27, 27%
CC	*n* = 13, 13%

**Table 2:** Comparison of MMP-1 and MMP-3 (mean ± S.D) among different epithelial ovarian carcinoma stages and histological subtypes by ANOVA test [F-value] and *p*-value.

Stages	Mean ± S.D of *MMP-1* (ng/mL)	Mean ± S.D of *MMP-3* (ng/mL)
Stage I [*n* = 30]	4.71 ± 0.73	36.18 ± 1.14
Stage II [*n* = 43]	6.06 ± 0.96	37.12 ± 1.35
Stage III [*n* = 15]	6.2 ± 0.90	37.56 ± 1.04
Stage IV [*n* = 12]	6.61 ± 0.82	37.7 ± 0.86
F-test, *p*-value	F = 21.14, *p* = 0.000	F = 7.23, *p* = 0.000
Histological subtype	Mean ± S.D of *MMP-1* (ng/mL)	Mean ± S.D of *MMP-3* (ng/mL)
SC, *n* = 45	5.6 ± 1.0	36.83 ± 1.63
MC, *n* = 15	5.38 ± 1.09	36.24 ± 1.55
EC, *n* = 27	5.8 ± 0.92	37.23 ± 1.33
CC, *n* = 13	5.5 ± 0.99	36.8 ± 1.5
F-test, *p*-value	F = 0.749, *p* = 0.520	F = 1.355, *p* = 0.261

**Table 3:** Distribution of *MMP-1 (-1607; 1G/2G)* and *MMP-3 (-1171; 5A/6A)* genetic variants in epithelial ovarian carcinoma stages.

	Tumor Stages							
MMPs Genotype Patients = 100	Stage 1, *n* = 30 *n*%		Stage 2, *n* = 43 *n*%		Stage3, *n* = 15 *n*%		Stage 4, *n* = 12 *n*%	
*MMP-3 [5A/6A]*								
*5A/5A*	7	23%	9	21%	3	20%	2	17%
*5A/6A*	17	57%	19	44%	7	47%	4	33%
*6A/6A*	6	20%	15	35%	5	33%	6	50%
χ^2^	20.97							
*p*	0.002							
*5A allele*	31	52%	37	43%	13	43.3%	8	33%
*6A allele*	29	48%	49	57%	17	56.7%	16	67%
χ^2^	7.33							
*p*	0.061							
*MMP-1 [1G/2G]*								
*1G/1G*	9	30%	12	28%	4	27%	2	17%
*1G/2G*	16	53%	21	49%	6	40%	4	33%
*2G/2G*	5	17%	10	23%	5	33%	6	50%
χ^2^	29.067							
*p*	0.000							
*1G allele*	34	57%	45	52%	14	47%	8	33%
*2G allele*	26	43%	41	48%	16	53%	16	67%
χ^2^	12.83							
*p*	0.005							

**Table 4:** *MMP-3 (5A/6A)* and *MMP-1 (1G/2G)* genotype and allele distribution in controls and in patients with different epithelial ovarian carcinoma histological subtypes.

	Carcinoma 100					Controls 60
MMP Genotypes	Histological Subtypes					
	All patients *n*%	SC = 45 *n*%	MC = 15 *n*%	EC = 27 *n*%	CC = 13 *n*%	*n*%
*MMP-3* *[5A/6A]*						
*5A/5A*	32 32%	17 38%	3 20%	8 30%	4 31%	39 65%
*5A/6A*	46 46%	23 51%	4 27%	13 48%	6 46%	15 25%
*6A/6A*	22 22%	5 11%	8 53%	6 22%	3 23%	6 10%
χ^2^	16.26 *	11.826 **				
*p*	0.000	0.066				
*5A allele* carrier	110 55%	57 63%	10 33%	29 54%	14 59%	93 77.5%
*6A allele*	90 45%	33 37%	20 67%	25 46%	12 46%	27 22.5%
χ^2^	16.370 *	18.266 **				
*p*	0.000	0.04				
OR [95% CI]	0.355 [0.213–0.593] *	2.0 [1.387–22.883] **				
*MMP-1 [1G/2G]*						
*1G/1G*	25 25%	14 31%	2 13%	7 26%	21 5.4%	42 70%
*1G/2G*	52 52%	22 49%	10 67%	14 52%	64 6.1%	14 23%
*2G/2G*	23 23%	9 20%	3 20%	6 22%	53 8.5%	4 7%
χ^2^	31.539 *	4.36				
*p*	0.000	0.620				
1G allele	102 51%	50 55%	14 47%	28 52%	10 38.5%	98 81.6%
2G allele	98 49%	40 45%	16 53%	26 48%	16 61.5%	22 18.4%
χ^2^	30.1 *	2.62 **				
*p*	0.000	0.453				
OR [95% CI]	0.23 [0.136–0.401] *	2.673 [1.786–4.0]				

* Comparison of genotype distribution between patients and controls. ** Comparison of genotype distribution among histological ovarian carcinoma subtypes.

**Table 5:** The *MMP-1* and *MMP-3* levels in different genotypes in controls and patients with epithelial ovarian carcinoma.

Parameters	*MMP-1* [ng/mL]				*MMP-3* [ng/mL]			
	*1G/1G*	*1G/2G*	*2G/2G*	ANOVA [F-Value] *p*-Value	*5A/5A*	*5A/6A*	*6A/6A*	ANOVA *p*-Value
Controls [*n* = 60]	[*n* = 42]	[*n* = 14]	[*n* = 4]	F = 29.36	*n* = 39	*n* = 15	*n* = 6	F = 9.33
	2.12 ± 0.49	3.05 ± 0.5	3.42 ± 0.15	*p* = 0.000	34.8 ± 2.3	35.3 ± 1.37	36.7 ± 0.60	*p* = 0.01
All patients [*n* = 100]	[*n* = 27]	[*n* = 47]	[*n* = 26]	F = 90.25	*n* = 21	*n* = 47	*n* = 32	F = 9.32
	4.35 ± 0.60	5.7 ± 0.64	6.50 ± 0.53	*p* = 0.000	36.1 ± 1.50	36.59 ± 1.60	37.6 ± 0.80	*p* = 0.000
Stage I [*n* = 30]	*n* = 9	*n* = 16	*n* = 5	F = 6.02	*n* = 7	*n* = 17	*n* = 6	F = 6.84
	4.37 ± 0.57	4.67 ± 0.47	5.5 ± 0.34	*p* = 0.007	35.4 ± 1.11	36.7 ± 1.2	37.7 ± 1.23	*p* = 0.004
Stage II [*n* = 43]	*n* = 12	*n* = 21	*n* = 10	F = 40.7	*n* = 9	*n* = 19	*n* = 15	F = 8.29
	4.88 ± 0.78	6.29 ± 0.44	6.98 ± 0.48	*p* = 0.000	35.8 ± 1.94	36.08 ± 1.70	37.95 ± 0.63	*p* = 0.001
Stage III [*n* = 15]	*n* = 4	*n* = 6	*n* = 5	F = 13.28	*n* = 3	*n* = 7	*n* = 5	F = 5.716
	5.17 ± 0.48	6.06 ± 0.64	7.16 ± 0.56	*p* = 0.001	36.04 ± 0.35	37.01 ± 0.63	37.73 ± 0.86	*p* = 0.018
Stage IV [*n* = 12]	*n* = 2	*n* = 4	*n* = 6	F = 7.07	*n* = 2	*n* = 4	*n* = 6	F = 59.23
	5.56 ± 0.31	6.23 ± 0.43	7.22 ± 0.71	*p* = 0.014	36.3 ± 0.07	36.81 ± 0.22	37.9 ± 0.22	*p* = 0.000

**Table 6:** Validity of *MMP-1,3* and CEA for differentiation between EOC patients and controls.

	AUC	SE	*p*-Value	CI	Sensitivity	Specificity
*MMP-3*	0.816	0.034	0.000	0.749–0.883	80.0%	75.3%
*MMP-1*	0.956	0.15	0.000	0.926–0.986	94.0%	90.0%
CEA	0.64	0.062	0.032	0.52–0.76	55.6%	61.6%

AUC: area under the curve; SE: standard error; CI: confidence interval.

**Table 7:** Validity of *MMP-1,3*, and CEA for differentiation between EOC stages I and II and stages III and IV.

	AUC	SE	*p*-Value	CI	Sensitivity	Specificity
*MMP-3*	0.797	0.043	0.000	0.713–0.80	77.8%	70.0%
*MMP-1*	0.806	0.43	0.000	0.721–0.890	74.1%	72.6%
CEA	0.703	0.059	0.002	0.588–0.819	62.3%	70.8%

AUC: area under the curve; SE: standard error; CI: confidence interval.

## Data Availability

Due to participant consent agreements, the data collected and analyzed in this study are not publicly available. However, the corresponding author may access them upon reasonable request.
